# An atypical presentation of a pleomorphic xanthoastrocytoma in a 66-year-old woman, a case report

**DOI:** 10.1016/j.radcr.2025.10.070

**Published:** 2025-11-18

**Authors:** Thomas Saliba, Ekkehard Hewer, Vincent Dunet

**Affiliations:** a Radiology department, Centre Hospitalier Universitaire Vaudois (CHUV), Lausanne, Switzerland; b Institute of Pathology, Lausanne University Hospital and University of Lausanne, Lausanne, Switzerland

**Keywords:** Tumor, Brain, Occipital, Glioma, MRI, Astrocytoma

## Abstract

Pleomorphic xanthoastrocytoma (PXA) is a rare astrocytic tumour, typically affecting young patients and most often located in the temporal lobe. We report an unusual case in a 66-year-old woman presenting with memory disturbance, dysphasia, headache, right-sided hemianopsia, and unilateral blindness. MRI imaging revealed a haemorrhagic, multiloculated, predominantly cystic lesion with extensive perilesional oedema in the left occipital lobe. Due to its atypical and non-specific appearance, the lesion could not be confidently diagnosed pre-operatively. Complete surgical resection was performed, and histopathology confirmed PXA, CNS WHO grade 2, with BRAF V600E mutation. The patient recovered without postoperative complications and remains disease-free at 1-year follow-up without adjuvant therapy. This case expands the known clinical and radiological spectrum of PXA, highlighting that it can occur in older patients, in uncommon locations, and with atypical imaging features. It underscores the importance of histopathological and molecular analysis for definitive diagnosis and supports gross total resection as the mainstay of treatment.

## Introduction

Pleomorphic xanthoastrocytomas (PXA) are astrocytic tumours that are hypothesised to originate from a line of subpial astrocytes, being first described in 1973 [[1], [2], [3]]. PXAs were first included in the 1993 WHO brain tumour classification system in 1993 as a grade 2 tumour, though later classifications would include anaplastic xanthoastrocytomas as grade 3 tumours [1,4]. They are very rare, making up less than 0.3% of primary central nervous system tumours and less than 1% of astrocytic tumours, with an annual incidence of < 0.7/100 000 people [5,6]. It is thought that, owing to the many different natural histories, PXAs may represent a group of molecularly different tumours, leaving open the possibility of further sub classifications emerging in the future though they remain histologically similar [1].

PXAs have the highest incidence in patients in the second decade of life, with the average age of diagnosis being 26 years old [1,7]. However, the youngest patient ever diagnosed was aged 2 years old and the oldest was 86, showing a wide range of ages [3,8]. There is debate as to whether there is an increased incidence of the tumour in women, with some articles stating that there may be a male: female ratio of 1.9: 1, whilst others report no differences [6,9].

The symptoms of patients are classed as localisable or non-localisable. Localisable symptoms are linked to the anatomical structures affected by the tumour, with epileptic seizures being the most common presenting symptom [1,8,10]. Non-localisable symptoms are headaches, nausea, diplopia, somnolence and vomiting which result from raised intracranial pressure [1].

The most common location for the tumour is in the temporal lobe, though they have been known to disseminate via the cerebrospinal fluid to the spinal cord, especially as the disease progresses [1,10]. Other locations where they have been described include the thalamus, cerebellum, the eye, pineal gland and sella turcica [10,11].

We present the case of a highly atypical pleomorphic xanthoastrocytoma discovered in a 66-year-old woman presenting with neurological symptoms and blindness in 1 eye.

## Case report

A 66-year-old woman presented to the neurologist with a 6-week history of memory impairment, dysphasia, and headaches. She also reported progressive vision loss in her right eye over the preceding 3 months. The patient sought medical attention after experiencing a worsening, non-pulsatile headache localized to the left temporal region.

She described increasing difficulty with speech production and word finding, as well as recent problems performing simple mathematical tasks. She denied nausea, vomiting, fever, photophobia, phonophobia, or other focal neurological symptoms.

On examination, her physical and neurological assessments were unremarkable. A prior MRI had revealed a left parietal lesion, previously suspected to represent a cavernoma.

Her past medical and surgical history were otherwise unremarkable. Her family history was also unremarkable. She was in good general health and was not taking any medications. The patient was known to be allergic to iodine-based contrast agents, though the severity of these reactions was not documented.A blood draw was not performed as it was not judged to be necessary given the clinical presentation of the patient.

**Fig. 1:**
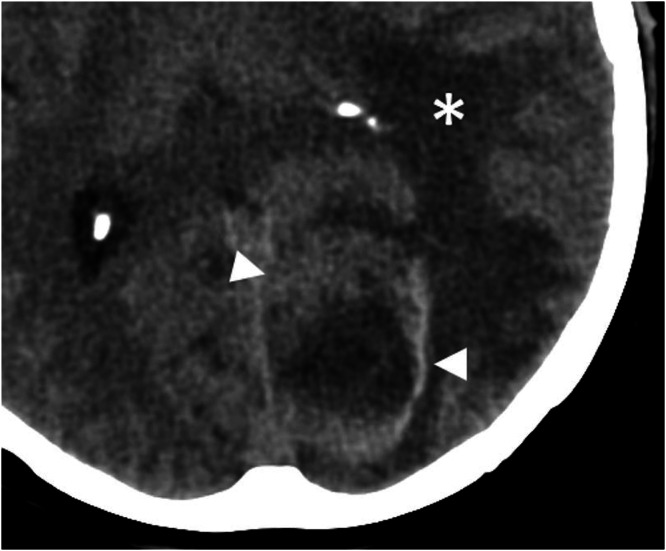
Pre-operative non-contrast CT with an axial reconstruction showing hypodense perilesional oedema (star) and hyperintense haemorrhage (arrowheads) surrounding an occipital lesion.

A CT was requested to investigate the new symptoms, with a suspicion of possible bleeding originating from the previously known the lesion. This revealed a haemorrhagic, non-calcified, lesion with perilesional oedema in the left occipital lobe (Fig. 1).

**Fig. 2:**
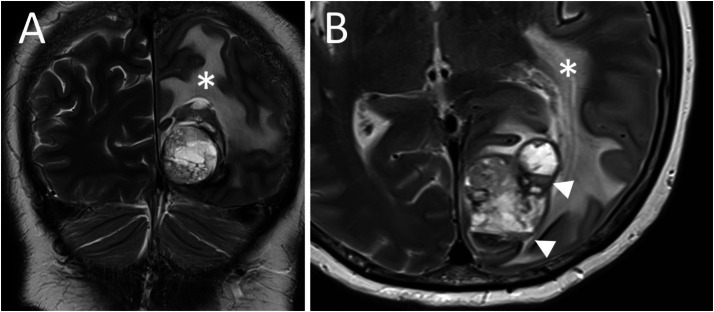
T2-weighted coronal (A) and axial (B) MRI images showing extensive perilesional oedema (star) and a multiloculated cystic occipital mass with fluid-fluid levels (arrowheads).

**Fig. 3:**
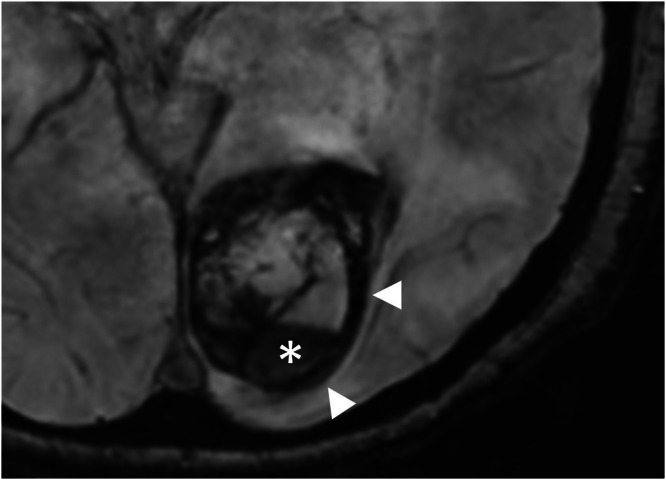
Susceptibility-weighted axial MRI image showing hypointense hemosiderin deposits surrounding the lesion (arrowheads) and a haemorrhagic fluid-fluid level (star).

**Fig. 4:**
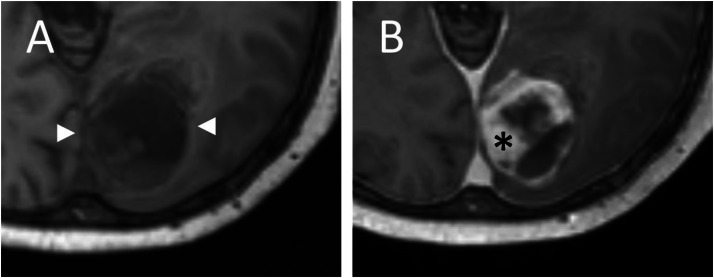
Non-contrast (A) and contrast-enhanced (B) axial T1-weighted imaging showing a heterogenous hypointense mass (arrowheads) with heterogenous enhancement (star) after contrast injection.

An MRI was ordered, revealing a left occipital lesion with extensive perilesional oedema (Fig. 2). The lesion appeared to be multiloculated and mainly cystic on T2-weighted imaging. The susceptibility weighted imaging confirmed the haemorrhage that had been seen on the CT scan, with the appearance of fluid-fluid levels of precipitated blood (Fig. 3). The lesion was heterogeneously isointense to the grey matter on T1-weighted imaging, presenting avid heterogenous enhancement after gadolinated contrast media injection (Fig. 4). The mass was in contact with the leptomeningeal surface but did not have a dural tail. Due to the non-specific characteristics, no definite pre-operative diagnosis was possible based on the imaging beyond confirmation of the likely tumoral nature of the lesion.

The patient was treated with steroids to reduce the inflammation as the operation was planned. One month after the initial diagnosis, the patient underwent an occipital craniotomy to allow the complete resection of the lesion.

Histopathological analysis showed a moderately glial neoplasm with pleomorphic tumor cells with often abundant eosinophilic cytoplasm. Eosinophilic granular bodies and occasional Rosenthal fibres were present. Focally, numerous hemosiderin-laden macrophages were present. No mitotic activity or necrosis were identified. Immunhistochemically, tumor cells were positive for glial fibrillary acidic protein (GFAP), CD34 V600E-mutant BRAF, and CD34, negative for R132H-mutant IDH1. A diagnosis of pleomorphic xanthoastrocytoma, CNS WHO grade 2 was issued. Methylation analysis with the Heidelberg classifier (v12.8) confirmed this diagnosis. The patient did not suffer any postoperative complication and was discharged in the days following the operation. As the resection was deemed to be complete, no further treatment with radiotherapy or chemotherapy was performed.

The patient continues to undergo regular radiological and clinical follow-ups with no signs of disease recurrence at 1-year post-surgery.

## Discussion

PXAs are rare tumours, mostly occurring in the second decade of life [1,7]. The tumour is nearly exclusively supratentorial, representing 98% of cases, and is most likely to affect the temporal lobes [11]. The higher prevalence in the temporal lobes can explain the seizures which are often the first symptom that prompts imaging [11]. They are also less commonly found in the parietal lobe and frontal lobe [9]. Our patient is atypical in that the lesion was located in the occipital lobe, which represents only about 7%-8% of cases [12,13].

PXAs can have a range of appearances, some being mainly solid masses, whilst others present as cystic structures with mural nodules [10,14]. PXAs have mixed attenuation on CT, with the low attenuation areas appearing as hyperintense on T2-weighed MRI imaging, representing cystic components [10,14]. The solid components show marked post-contrast enhancement on CT, being isointense to the grey matter on T1-weighted imaging and hyperintense to the grey matter on T2-weighted imaging [3,10]. These solid components will also enhance on post-contrast MRI imaging [3,10]. In some patients, there may be enhancement around the cystic components as well as leptomeningeal enhancement, though it is relatively rare as it occurs in around 14% of cases, compared to leptomeningeal contact which is evident in 92% of lesions [6,10,14]. Diffusion imaging can be slightly hyperintense to surrounding tissue, with ADC values being slightly increased [14]. There may also be perilesional vasogenic oedema, which appears as hyperintense in T2-weighed imaging, which is found in around 80% of patients [10,15]. Another feature, present in 40% of patients in 1 case series, is calcifications [3,10]. Inner table remodelling with lytic lesions and scalloping may also be present but it is relatively rare and non-specific [6,10]. Some lesions can also appear to have a dural tail, mimicking meningiomas [10,14].

The main imaging differential diagnosis is gliomas, though PXAs have significantly less hypercellularity and peritumoral oedema, nevertheless it is extremely difficult to differentiate between the different families tumours [6,16]. Other differential diagnoses are ganglioglioma, pilocytic astrocytomas, dysembrionoplastic neuroepithelial tumours and meningiomas [6]. PXA can be differentiated from gangliogliomas due to being less likely to be calcified and having less peritumoral oedema [6]. Location can provide a clue for differentiating PXA from pilocytic astrocytomas, as the latter are more likely to occur in the infratentorial region, compared to the nearly exclusive supratentorial location of PXA [6].

Our patient’s case is highly unusual. PXAs are seldom found in women in their Seventh decade of life. Furthermore, the occipital location and large amount of perilesional oedema are not common features of PXAs. The post-contrast enhancement is, however, typical. The rest of the characteristics are compatible with PXAs but are non-specific and thus do not help in narrowing the differential diagnosis.

PXA is classified as a grade II according to the WHO, being characterised by nuclear atypia alone [11]. The typical histopathology of PXAs include pleomorphism, multinucleated giant cells, giant cells with nuclear atypia and foamy tumoral cells [17]. In cases where there are ≥ 5 mitoses/10 high power field, the tumour is classified as an anaplastic pleomorphic xanthoastrocytoma, a grade III tumour according to the WHO, with a much more dire prognosis [17]. As the morphology, location and immunological markers are similar to PXA, pathology is essential in establishing the differential diagnosis [17].

Surgical resection is the main treatment for PXAs, with debate around the utility of postoperative radiotherapy and chemotherapy due suboptimal results, especially in male patients [10,18]. One report found that radiotherapy resulted in increased mortality rates in adult patients and another study found that there was no survival benefit when patients received chemotherapy [11]. However, recent studies indicate that patients with PXAs exhibiting BRAF V600 mutations, present in 70% of tumours, may benefit from BRAF inhibitors such as vemurafenib or dabrafenib, though more studies are needed [11,19].

Older patients have worse survival rates, with 1 study suggesting that 39 years should be used a cut-off and that more aggressive treatment is warranted as the patients are considered higher risk [18]. Another factor is tumour size, as patients with PXAs over 30mm have a worse prognosis [18]. Nevertheless, the prognosis remains favourable, with a 75% 5-year survival and 67% ten-year survival rate, with full resection being the main determining factor [7,11].

## Conclusion

We describe a rare pleomorphic xanthoastrocytoma in an elderly woman with occipital involvement and extensive oedema, features seldom reported in the literature. It broadens the recognized clinical and radiological spectrum of PXAs and underscores the diagnostic challenge when presentation deviates from the typical young patient and temporal lobe location. By highlighting the role of histopathology and molecular testing in confirming diagnosis, this report reinforces the need to keep PXA in the differential for atypical supratentorial tumours and supports complete surgical resection as an effective treatment option.

## Patient consent

The patient provided written consent for their data to be used for a case report.
